# The Influence of Mobile Health Interventions on Aftercare and Medication Use for Patients With Chronic Pain: A Systematic Review

**DOI:** 10.1016/j.mcpdig.2026.100364

**Published:** 2026-04-27

**Authors:** Tom W.A. Neetens, Bart Hiemstra, Gitara M. Edward, Rudolf B. Kool, Margot H.J. Roozekrans, Markus W. Hollmann, Thomas Timmers

**Affiliations:** a Department of Anaesthesiology and Pain Medicine, Noordwest Ziekenhuisgroep, Alkmaar, Netherlands; b Department of Anaesthesiology and Pain Medicine, Amsterdam UMC Location AMC, Amsterdam, Netherlands; c Radboud University Medical Center, Radboud Institute for Health Sciences, IQ Healthcare, Nijmegen, Netherlands; d IQ Health Science Department, Radboud University Medical Center, Nijmegen, Netherlands

## Abstract

**Objective:**

To evaluate the impact of mobile health interventions (MHIs) on opioid use, nonopioid medication use, adherence, quality-adjusted life years (QALYs), and health care utilization among adults with chronic pain.

**Patients and Methods:**

A systematic review (PROSPERO CRD4202457819) was conducted using PubMed, Embase, CINAHL, Web of Science, and the Cochrane Library from April 23, 2024, to May 2, 2025. Randomized controlled trials (RCTs) evaluating mobile-accessible interventions for adults aged 18-65 years with chronic pain, low back pain, or chronic low back pain were included. Two reviewers independently screened studies and assessed risk of bias. Owing to heterogeneity, outcomes were synthesized narratively, and standardized mean differences were calculated when appropriate.

**Results:**

Fourteen trials (n=3766) met inclusion criteria. Six studies (n=1098) evaluated opioid use, with 4 reporting significant reductions. All 5 studies assessing nonopioid medication use (n=810) demonstrated decreases. Of 4 studies examining adherence (n=851), 3 showed improvement. QALY gains were observed in 4 of 5 studies (n=2252), although effect sizes were small. No significant reductions in health care utilization were identified across included trials.

**Conclusion:**

Mobile health interventions reduce opioid and nonopioid medication use and improve adherence in adults with chronic pain. Evidence for QALY improvement is modest, and effects on health care utilization remain uncertain. Moreover, MHIs serve as valuable adjuncts to chronic pain care, particularly for medication stewardship. Further high-quality, long-term randomized trials using standardized outcome measures are needed.

**Article Highlights**

• Mobile health interventions (MHIs) reduce opioid and nonopioid medication use in patients with chronic pain.
• MHIs improve adherence to treatment and/or medication across diverse chronic pain populations.
• Quality-adjusted life year gains from MHIs are small but consistently positive.
• No clear evidence supports reductions in health care utilization.
• Mobile health interventions function as valuable adjuncts to standard care, particularly for medication stewardship.

Chronic pain (CP) is a growing global health concern, affecting individuals across all demographic characteristics and placing a considerable burden on both patients and health care systems.^1^ Chronic pain substantially diminishes quality of life, limiting physical function, emotional well-being, and social participation.^2^ Beyond persistent discomfort, it often leads to sleep disturbance, mood disorders, and reduced ability to engage in work and daily activities.^2^

A substantial portion of CP is represented by low back pain (LBP). According to the World Health Organization (WHO), most people will experience LBP at 1 point in their life, and in 2020, 619 million people were actively experiencing LBP.^3^ Chronic LBP (CLBP) is defined by a period of at least 12 weeks in which back pain symptoms are continuously present. About two-thirds of the patients experiencing CP have CLBP.^4^ In the United States, yearly CLBP-related health care costs are estimated to range between 12 and 90 billion dollars.^5^

Opioid analgesics have historically played an important role in the management of CP, particularly when other treatments fail to provide adequate relief. However, long-term opioid therapy for chronic noncancer pain is increasingly discouraged owing to risks such as dependence, tolerance, overdose, and limited long-term effectiveness.^2^^,^^6^, ^7^, ^8^, ^9^ Consequently, many international guidelines now emphasize nonpharmacologic treatment strategies and careful opioid stewardship. For this reason, changes in opioid prescribing and medication use are frequently used as important outcome measures in CP research.

Managing CP often requires frequent interactions between patients and health care providers, both during periods of medication titration and nonpharmacologic treatments.^2^ These patient-provider interactions consume substantial time and resources and contribute to increasing health care workloads.^4^ In addition, many health care systems are experiencing increasing workforce pressures owing to aging populations, rising prevalence of chronic diseases, and shortages of health care professionals or funding.^10^^,^^11^ These challenges have stimulated interest in alternative care delivery models that may improve efficiency while maintaining quality of care.

Given the rising demand for CP care and the growing scarcity of health care professionals, there is an urgent need to explore more efficient methods of care delivery. Digital health technologies, including mobile health interventions (MHIs), have therefore attracted increasing interest as potential tools to support chronic disease management.^10^ Mobile health interventions broadly refer to health services or interventions delivered through mobile or internet-based technologies such as smartphone applications, web platforms, messaging systems, or telemonitoring tools.

In CP care, these interventions may support patient education, behavioral change, exercise programs, symptom monitoring, and communication with health care providers.^12^, ^13^, ^14^, ^15^, ^16^ Such approaches may not only enhance patient engagement and self-management but also allow clinicians to monitor patient progress remotely. Previous research has suggested that MHIs can improve patient engagement and various clinical outcomes, but evidence regarding their impact on medication use, adherence, and health care utilization remains heterogeneous.^17^^,^^18^ This systematic review aims to assess the impact of MHI on aftercare and medication use in patients with CP, with a focus on improving both care efficiency and patient outcomes.

## Patients And Methods

### Study Design

This is a systematic review of randomized controlled trials (RCTs) assessing the effects of MHIs on aftercare and medication use in patients with CP.

#### Definition of MHIs

For the purpose of this review, MHIs were defined as interventions that used digital or mobile technologies to deliver components of CP management, including patient education, behavioral therapy, monitoring, or self-management support. Eligible delivery platforms included smartphone applications, web-based platforms accessible through mobile devices, short message service (SMS) messaging systems, telemonitoring tools, and interactive voice response systems.

Video consultations and telehealth components were included when they were embedded within a broader digital intervention aimed at supporting patient self-management or remote monitoring. This definition was chosen because many contemporary digital health programs combine several digital modalities rather than relying on a single technological tool.

#### Population Definition

Eligible studies included adult patients aged 18-65 years with CP, CLBP, or LBP where the majority of participants were reported to have persistent symptoms consistent with CP. When studies included broader LBP populations, we assessed whether the trial population predominantly represented chronic or persistent pain conditions (eg, symptom duration ≥ 12 weeks or recruitment from CP services). Studies focusing exclusively on acute LBP were excluded.

### Search Strategy and Selection Criteria

We performed a comprehensive literature search in 5 databases: PubMed, Embase, CINAHL, Web of Science, and the Cochrane Library. The initial search was conducted on April 23, 2025, with an updated search on May 2, 2025, to capture recently published articles. The search strategy incorporated synonyms and Boolean operators (detailed for PubMed in Supplemental Appendix 1, available online at https://www.mcpdigitalhealth.org/). Only studies published in 2000 and later were included. There were no language restrictions. We did not assess gray literature or trial registries. If only an abstract was available, we attempted to retrieve the full text by contacting the study authors. In addition, reference lists of included articles were screened (snowballing) to identify potentially relevant studies not captured in the initial searches. When needed, we contacted study authors for unpublished data.

Eligible studies were RCTs investigating MHI targeting adult patients (aged 18-65 years) with CP, LBP, or CLBP. Interventions had to be accessible via mobile devices and could include video conferencing, applications, SMS, or web-based platforms. Exclusion criteria included studies on pain unrelated to the lower back (except general CP), cancer pain, nondigital interventions, or ineligible designs (eg, reviews and editorials).

Two reviewers (T.W.A.N. and B.H.) independently screened all titles, abstracts, and full text articles. Discrepancies were resolved with a third reviewer. The review protocol was registered with PROSPERO (CRD4202457819).

### Data Analysis (by Outcome)

Owing to heterogeneity in study design, study populations, interventions, and outcomes, meta-analysis was not feasible. In the included studies, all outcomes regarding aftercare and medication use were identified. The frequency of occurrence of each outcome was assessed. The 5 most prominent outcomes were selected for analysis. To enable comparison of intervention effects across the included studies, standardized mean differences (SMDs) with corresponding 95% CIs were calculated. The effect size was interpreted in line with Cohen benchmarks, categorizing values as small (more than −0.2 to ≤0.2), moderate (>0.2 to <0.8), or large (≥0.8).^19^ Depending on the outcome, either a positive or a negative SMD may represent a favorable effect. Consequently, both positive and negative SMD values were retained in the analyses.

## Results

### Included Studies

**Figure 1:**
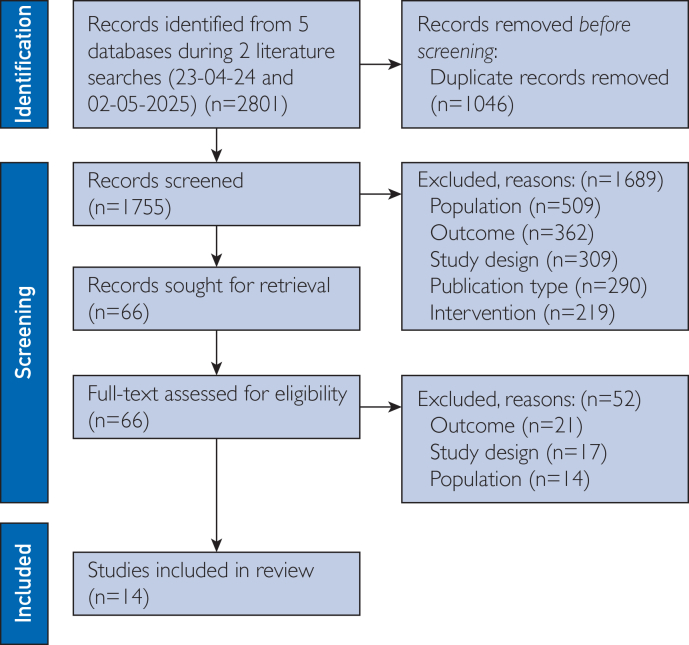
PRISMA (Preferred Reported Items for Systematic Reviews and Meta-Analyses) flow diagram of literature searches.^20^

After searching 5 databases, a total of 2801 studies were found (Figure 1).^20^ After duplicates (1046) were removed, 1755 studies were screened on title and abstract. During this process, 1689 studies were excluded, leaving 66 studies to be assessed for eligibility. Finally, 52 studies were excluded, leaving a total of 14 studies that were included in this review: 9 RCTs, 2 cost-effectiveness analyses of RCTs, 2 pilot RCTs, and 1 feasibility RCT. Six studies were conducted in the United States, 4 studies in the United Kingdom, 2 studies in Australia and singular studies were performed in Denmark and the Netherlands. An overview and the characteristics of the included studies can be found in Table 1.^21^, ^22^, ^23^, ^24^, ^25^, ^26^, ^27^, ^28^, ^29^, ^30^, ^31^, ^32^, ^33^, ^34^

**Table 1:** Overview of the Included Studies

Reference, year	Country	Design	Participants	Intervention	Outcomes aftercare	Outcomes and medicine use
Cooperman et al,^21^ 2024	United States	RCT	154; adults with CP and opioid use disorder	Video conferencing	—	Opioid use Nonopioid use Adherence
Wilson et al,^22^ 2023	United States	RCT	402; adults with CP	Online program	—	Opioid use Adherence
Kroenke et al,^23^ 2014	United States	Cost-effectiveness analysis of RCT	250; adult veterans with chronic musculoskeletal pain	Online or interactive voice recorded telephone calls	Health care utilization	Opioid use Nonopioid use
Gholamrezaei et al,^24^ 2024	Australia	Pilot RCT	26; adults with CP undergoing opioid tapering	Video and text messages	—	Opioid use
Odineal et al,^25^ 2020	United States	RCT	215; adults with CP	Mobile application	—	Opioid use Nonopioid use
Naylor et al,^26^ 2010	United States	RCT	51; adults with CP	Interactive telephone-based voice response	—	Opioid use Nonopioid use
MacFarlane et al,^27^ 2021	United Kingdom	RCT	996; adults at high risk of chronic widespread pain	Telephone cognitive behavioral therapy	QALY	—
Geraghty et al,^28^ 2018	United Kingdom	Feasibility RCT	87; adults with LBP	Internet intervention or internet intervention plus telephone support	QALY	Adherence
Fatoye et al,^29^ 2020	United Kingdom	RCT	47; adults with nonspecific CLBP	Mobile application	QALY	—
Geraghty et al,^30^ 2024	United Kingdom	RCT	825; adults with LBP	Internet intervention or internet intervention plus telephone support	QALY	—
Kongstad et al,^31^ 2024	Denmark	Cost-effectiveness analysis of RCT	297: adults with LBP	Mobile application	QALY	—
Tankha et al,^32^ 2024	United States	RCT	140; adults with CLBP	(Live) streaming	—	Nonopioid use
Koppenaal et al,^33^ 2022	Netherlands	RCT	208; adults with LBP	Mobile application	—	Adherence
Amorim et al,^34^ 2019	Australia	Pilot RCT	68; adults with CLBP	Mobile application	Health care utilization	—

CLBP, chronic low back pain; CP, chronic pain; LBP, low back pain; QALY, quality-adjusted life year; RCT, randomized controlled trial.

### Patient Population

All patient populations were adults with noncancer pain. Seven of the included studies discussed findings in a patient population with general CP. In 4 of the included studies patients with LBP were targeted and another 3 studies targeted patients with CLBP. Two studies focused on patients using opioids, and in 1 study, the patient population consisted of veterans with chronic musculoskeletal pain (Table 1).

### Risk of Bias of Included Studies

**Figure 2:**
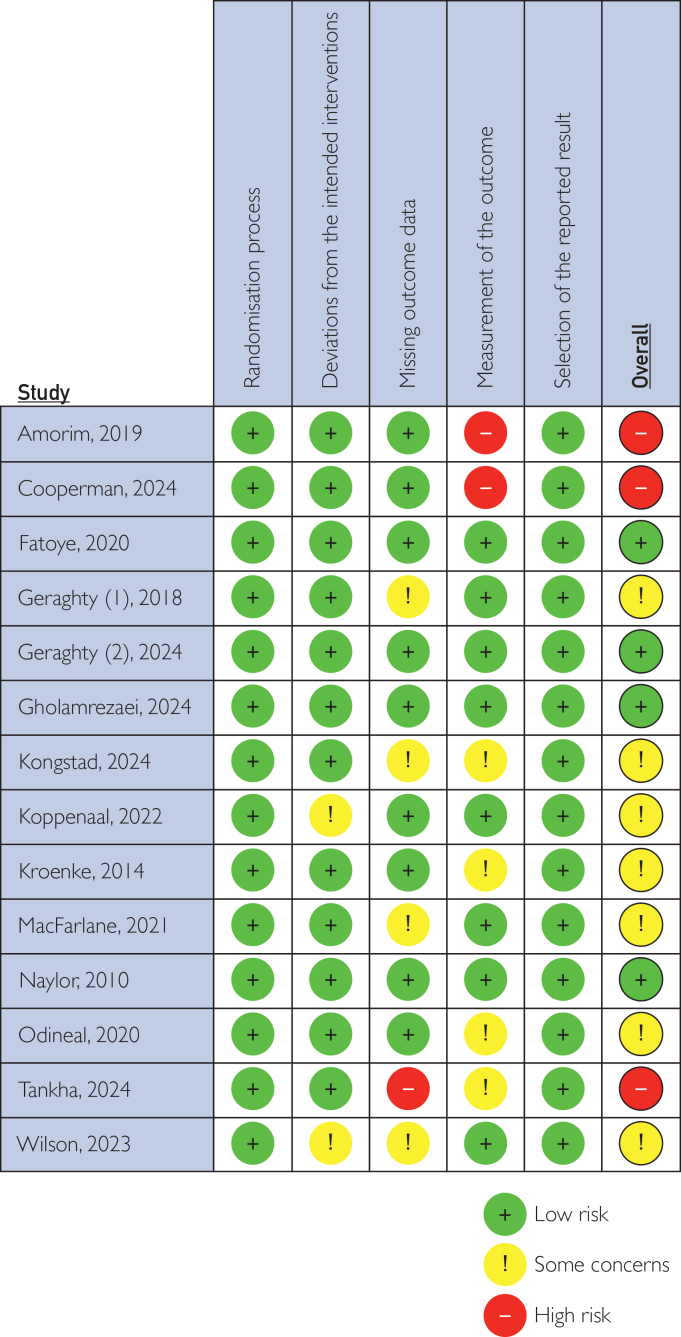
Risk of bias assessment using the Cochrane Collaboration’s tool for assessing risk of bias in randomized trials.^35^

Two reviewers (T.W.A.N. and B.H.) independently assessed all 14 trials for risk of bias. Discrepancies were resolved with a third reviewer. Trials were assessed for risk of bias on 5 domains: randomization process, deviations from the intended interventions, missing outcome data, measurement of the outcome, and selection of the reported result. For the interpretation of individual study findings, the methodological quality should be considered. Of the 6 studies assessing opioid use, 2 were rated as low risk of bias, 3 as having some concerns, and 1 as high risk of bias. The studies showing significant reductions in opioid use were primarily those with low or moderate risk of bias, although sample sizes varied considerably (Figure 2).^35^

### Outcomes

The 5 most prominent outcomes and their corresponding frequency were as follows: opioid use (6 studies, 1098 patients); quality-adjusted life years (QALYs; 5 studies, 2252 patients); nonopioid medicine use (5 studies, 810 patients); adherence (to medication and/or intervention; 4 studies, 851 patients); and health care utilization (2 studies, 318 patients). The studies evaluating QALYs and health care utilization were selected for further analysis regarding the influence on aftercare. For the influence on medicine use the studies regarding opioid use, nonopioid medicine use, and adherence to intervention were selected. An overview of the studies and the corresponding outcomes can be found in Table 1.

#### Opioid Use

Six studies investigated opioid use as an outcome of MHI (Table 2). A total of 1098 patients were included across these studies; MHI incorporating telehealth, self-monitoring, or psychoeducation were found to be associated with reductions in opioid use.

**Table 2:** Studies Assessing Opioid Use, Quality-Adjusted Life Years, Nonopioid Medicine Use, Adherence to Medication and/or Intervention, and Health Care Utilization

Reference, year	Measurement	After	Result	SMD (95% CI)
Opioid use				
Cooperman et al,^21^ 2024	Return to opioids	16 wk	+	−0.13 (−0.447 to 0.186)
	Days of opioid use	16 wk	+	−0.04 (−0.22 to 0.14)
Wilson et al,^22^ 2023	≥15% reduction in daily medications	6 mo	+	NA^a^
Kroenke et al,^23^ 2014	Months of opioid use	12 mo	−	−0.1 (−0.348 to 0.148)
Gholamrezaei et al,^24^ 2024	Opioid tapering self-efficacy scale	4 wk	+	0.69 (−0.13 to 1.52)
Odineal et al,^25^ 2020	Morphine milligram equivalents	6 mo	−	NA^b^
Naylor et al,^26^ 2010	Mean dose change	8 mo	+	NA^a^
Quality-adjusted life years				
MacFarlane et al,^27^ 2021	EQ-5D-5L	24 mo	+	0.13 (0.04-0.22)
Geraghty et al,^28^ 2018	EQ-5D-5L	3 mo	NA	NA^a^
Fatoye et al,^29^ 2020	SF-6D	8 mo	+	NA^b^
Geraghty et al,^30^ 2024	EQ-5D-5L	12 mo (MHI)	+	0.1 (−0.11 to 0.31)
	EQ-5D-5L	12 mo (MHI + telephone support)	+	−0.18 (−0.39 to 0.03)
Kongstad et al,^31^ 2024	EQ-5D	9 mo	+	0.03 (0.01-0.05)
Nonopioid medicine use				
Cooperman et al,^21^ 2024	Days of illicit drug use	16 wk	+	−0.44 (−0.49 to 0.40)
Kroenke et al,^23^ 2014	Months of analgesic use	12 mo	+	−0.69 (−0.94 to −0.43)
Odineal et al,^25^ 2020	Less NSAID prescribing	6 mo	+	NA^b^
Naylor et al,^26^ 2010	Less NSAID use	8 mo	+	0.31 (−0.24 to 0.86)
Tankha et al,^32^ 2024	Less NSAID use	24 wk	+	0.37 (0.04-0.70)
Adherence				
Cooperman et al,^21^ 2024	To methadon (self-reported)	16 wk	+	0.4 (0.06-0.74)
Wilson et al,^22^ 2023	To program (by attendance)	6 mo	+	NA^a^
Geraghty et al,^28^ 2018	To intervention (with or without telephone support)	3 mo	NA	NA^a^
Koppenaal et al,^33^ 2022	Exercise adherence rating scale	3 mo	−	0.31 (0.03-0.58)
Health care utilization				
Kroenke et al,^23^ 2014	Outpatient visits	12 mo	−	−0.02 (−0.27 to 0.22)
Amorim et al,^34^ 2019	Reduction in rate of care seeking	6 mo	−	NA^a^

+, significant benefit in favor of the intervention group; −, no significant benefit in favor of the intervention group; EQ-5D, EuroQol- 5 dimensions; EQ-5D-5L, EuroQol-5 dimensions- 5 Levels; MHI, mobile health intervention; NA, not available; NSAID, nonsteroidal anti-inflammatory drug; SF-6D, short form 6- dimensions; SMD, standardized mean difference.

a The SMD could not be calculated because the required data were unavailable despite repeated requests to the study authors.

b The SMD could not be calculated because the available data sets were incomplete.

One large RCT (n=402) evaluated an online pain self-management program designed to complement usual care. The program included educational modules about CP mechanisms, behavioral pain management strategies, medication safety information, and guided self-monitoring exercises. Participants receiving the intervention showed a greater proportion achieving ≥15% daily opioid dose reduction than the usual care group.^23^ Another study (n=154) showed a lower number of days of drug and opioid use in the intervention group over 16 weeks.^22^ During a pilot RCT (n=26), participants in the intervention group—who received an opioid tapering self-efficacy scale, a psychoeducational video, and SMS text messaging—achieved a greater reduction in opioid consumption during tapering than the control group.^25^

Overall, 4 studies found statistically significant reduction in opioid use.^22^^,^^23^^,^^25^^,^^27^ The remaining studies did not find a difference between intervention and control groups regarding opioid use.^24^^,^^26^ SMD were small in 2 studies^22^^,^^24^ and moderate in 1 study.^25^

#### Quality-Adjusted Life Years

Five studies reported on QALYs (Table 2). Across all studies, 2252 patients were included. Four of 5 studies reported small but positive QALY gains.^28^^,^^30^, ^31^, ^32^ The remaining study did not report QALY outcomes because the variability in cost data introduced substantial uncertainty into the cost-effectiveness estimates, making any calculation regarding QALYs unreliable.^29^

A population-based RCT (n=996) showed a mean increase of 0.023 QALYs at an incremental cost of £1828.^28^ Another large trial (n=825) showed that both forms of an internet intervention showed QALY gains and a greater cost-effectiveness than usual care.^31^ Moreover, SMDs were small in all 4 studies that reported QALY gains.^28^^,^^30^, ^31^, ^32^

#### Nonopioid Medicine Use

Five studies investigated the influence of MHI on nonopioid medicine use (Table 2). Across these studies, 810 patients were included. All interventions showed a significantly decreased use or prescription of nonopioid medicines.

In 1 study (n=215), participants in the intervention group showed significantly lower rates of NSAID prescription.^26^ In an RCT using mindfulness as digital intervention (n=154), the intervention led to a significant reduction in days of any drug use, including not only opioids but also other illicit drug use.^22^ SMD ranged from small in 1 study^16^ to moderate in 3 studies.^22^^,^^27^^,^^33^

#### Adherence (Medication and/or Intervention)

Four studies investigated the influence of mobile applications on adherence to treatment or intervention (Table 2). A total of 851 patients were included; MHI mostly showed a positive effect on medication and intervention adherence.

Significant improvements in adherence were observed in 3 studies.^22^^,^^23^^,^^34^ One study failed to report *P* values or CIs.^29^

One study (n=154) assessed the influence of mindfulness through videoconferencing, demonstrating a significantly higher methadone adherence at 16 weeks in the intervention group.^22^ A multicenter RCT (n=402) found that 68% of participants achieved a predefined threshold of substantive exposure to the online self-management program.^23^ A study (n=208) evaluating stratified blended physiotherapy adherence found improved home exercise adherence through a mobile application intervention.^34^

In a feasibility RCT (n=87), usual care was compared with 2 types of internet interventions (with or without telephone support). In this study, the effect of intervention adherence could not be determined owing to a lack of *P* values or CIs.^29^ Furthermore, SMDs were moderate in 2 studies.^22^^,^^34^

#### Health Care Utilization

Two studies assessed health care utilization (Table 2). These studies included 318 patients. Both studies did not report statistically significant differences in health care utilization.

In 1 trial (n=68), a 38% reduction in care seeking was observed in the intervention group compared with controls, but this was not statistically significant.^36^ The other study (n=250) found no significant differences in outpatient visits, emergency visits, or hospitalizations and SMD was small.^24^

## Discussion

This systematic review evaluates the influence of MHI for CP, including LBP and CLBP. In total, 14 RCTs were included in the analysis, encompassing 3766 patients. Through the studies, various modes of delivery were used, ranging from applications and online platforms to video and interactive voice response systems. Overall, the included studies suggest that MHIs may contribute to reductions in opioid and nonopioid medication use and improvements in treatment adherence. However, the strength of this evidence should be interpreted cautiously because several studies were assessed as having moderate or high risk of bias and considerable heterogeneity was present in both the interventions and outcome measures. Small effects were found for QALYs and no significant outcome was shown for health care utilization.

An important methodological consideration is the nature of the standard care provided in the control groups. Across the included trials, usual care varied considerably and ranged from primary care follow-up by general practitioners to multidisciplinary care in specialized pain management centers. In several studies, usual care consisted mainly of periodic consultations without structured behavioral or self-management support.

This variability makes it difficult to determine whether the observed benefits of MHIs are primarily attributable to the digital delivery format or to the additional patient engagement and self-management support embedded within the interventions. It is possible that MHIs partly compensate for limitations in standard care by providing continuous monitoring, educational materials, and behavioral guidance that may not always be feasible in routine clinical practice.

Health care guideline bodies increasingly recommend nonpharmacologic, person-centered, and self-management approaches for CP, while discouraging long-term opioid therapy.^6^, ^7^, ^8^, ^9^, ^10^ Our findings support these recommendations and clearly demonstrate the added value of using MHI in doing so: 4 of 6 trials assessing opioid use reported significant reductions—particularly where tapering and behavioral support were embedded,^22^^,^^23^^,^^25^^,^^27^ whereas all 5 studies on nonopioid analgesics observed decreased use.^22^^,^^24^^,^^26^^,^^27^^,^^33^ Three of 4 trials showed improved adherence to prescribed interventions or medicines,^22^^,^^23^^,^^34^ suggesting that the value of MHI lies less in direct pain reduction and more in medication stewardship and adherence. This interpretation complements WHO and National Institute for Health and Care Excellence recommendations prioritizing education, exercise, and psychological interventions.^11^

Economic and utility outcomes in our review showed modest QALY gains, sometimes associated with increased costs.^28^^,^^30^, ^31^, ^32^ This pattern mirrors recent cost-effectiveness analyses, indicating that MHI may deliver small but favorable benefits, although without consistent health care utilization savings.^24^^,^^35^^,^^36^

This review’s strengths include the exclusive use of RCTs, enhancing internal validity, and the inclusion of predominantly digital or telephone-based interventions, which facilitated low rates of missing outcome data. However, heterogeneity in interventions, populations (eg, opioid tapering cohorts vs broader CP), and outcome measures precluded meta-analysis and limits generalizability. Several trials relied on self-reported medication and adherence outcomes. However, self-reported outcomes are gathered directly from patients, without interference or external influences and doing so digitally, be a more time-effective way compared with, for instance, paper-based questionnaires.

Three studies were judged at high risk of bias, reducing confidence in pooled findings. Safety was rarely and inconsistently reported, and adverse events such as anxiety from monitoring, digital fatigue, and dropout reasons were underexplored.

Mobile health interventions should not be considered as a replacement but a valuable access to standard care, to improve adherence and ownership, without putting extra pressure on health care, or without denying patient’s personal care. Their greatest value appears to lie in medication stewardship and treatment adherence, consistent with WHO and National Institute for Health and Care Excellence guidance.^6^^,^^10^ Key features of potentially effective digital tools identified in the included studies concerned structured self-management modules (eg, education and graded activity), behavioral therapy components such as mindfulness or cognitive behavioral strategies, automated reminders and feedback systems, and integrated opioid tapering support tools.^10^^,^^13^^,^^22^^,^^23^^,^^25^^,^^34^

Patient selection is critical, and MHI may be best suited for patients undergoing opioid tapering, those with low-to-moderate complex clinical background, and high users where medication stewardship is a priority. Expectations should remain realistic, given modest QALY gains and uncertain reductions in health care utilization.^23^ Successful implementation of MHI requires attention to health equity and sociocultural factors, secure handling of health data, interoperability with existing electronic systems, and transparent evaluation of safety and effectiveness.^10^

Future research should focus on RCTs with 12 to 24 months follow-up or more, using standardized outcome sets that capture medication use, adherence, QALYs, and health care utilization.^14^ Component- and dose-finding studies, including head-to-head comparisons of cognitive behavioral therapy modules, personalization strategies, and clinician feedback intensity, are needed to identify active ingredients driving adherence and medication reductions.^13^ Pragmatic and hybrid effectiveness–implementation studies using frameworks such as Reach, Effectiveness, Adoption, Implementation, and Maintenance (RE-AIM) should evaluate adoption, costs, and feasibility in real-world contexts.^15^

Equity-focused analyses, including moderators such as baseline pain or disability, psychological factors, and digital literacy are essential, and more evidence from low- and middle-income country settings is required.^16^ Balance between patient characteristics is essential to accurately represent the CP patient population. In this review, for example, certain patient groups or geographic areas were represented more or less prominently. Safety and unintended consequences must be systematically reported using harmonized definitions, covering adverse events, digital fatigue, and discontinuation reasons.

## Conclusion

This systematic review suggests that MHIs may support reductions in opioid and nonopioid medication use and may improve adherence to treatment or self-management strategies in patients with CP. However, the certainty of the evidence remains limited owing to heterogeneity in study populations, interventions, and outcome measures, as well as varying risk of bias across studies. Small gains were witnessed in QALYs, and no clear evidence of reduced health care utilization was shown. Further high-quality, long-term trials with integrated economic and safety evaluations are needed to clarify their role in sustainable, guideline-concordant CP management.

## Potential Competing Interests

The authors report no competing interests.
