# Supplementary material for: The Influence of Mobile Health Interventions on Aftercare and Medication Use for Patients With Chronic Pain: A Systematic Review
Source: Mayo Clin Proc Digit Health. 2026 Apr 27;4(2):100364. doi: 10.1016/j.mcpdig.2026.100364 (PMC13223912; doi:10.1016/j.mcpdig.2026.100364)
Supplement: Supplemental Appendix [file mmc1.docx]

**Appendix A.** Medline (PICO-)Search Strategy, performed on 23-04-24 and 02-05-2025

("Neuralgia"[Mesh] OR Neuralgia*[tiab] OR Neuropathic Pain*[tiab] OR NeuP[tiab] OR Neurodynia*[tiab] OR Nerve Pain*[tiab] OR "Chronic Pain"[Mesh] OR Chronic Pain*[tiab] OR Persistent pain*[tiab] OR "Fibromyalgia"[Mesh] OR "Low Back Pain"[Mesh] OR Fibromyalg*[tiab] OR Muscular Rheumat*[tiab] OR Fibrositi*[tiab] OR Myofascial Pain Syndrom*[tiab] OR Low Back Pain*[tiab] OR Lumbago*[tiab] OR Lower Back Pain*[tiab] OR Low Back Ache*[tiab] OR Low Backache*[tiab] OR Low Backache*[tiab])

AND

("Computers, Handheld"[Mesh] OR Handheld Computer*[tiab] OR Tablet Computer*[tiab] OR Palmtop Computer*[tiab] OR Palm-Top Computer*[tiab] OR Personal Digital Assist*[tiab] OR PDA Computer*[tiab] OR Pocket PC*[tiab] OR Mobile Device*[tiab] OR Palm Pilot*[tiab] OR "Mobile Apps"[Mesh] OR Mobile Applicat*[tiab] OR Mobile App[tiab] OR Mobile Apps[tiab] OR Portable Apps[tiab] OR Portable App[tiab] OR Portable Applicat*[tiab] OR "Smartphone"[Mesh] OR Smartphone*[tiab] OR Smart Phone*[tiab] OR "Telemedicine"[Mesh] OR Telemedicine*[tiab] OR Tele-Referral*[tiab] OR Tele Referral*[tiab] OR Virtual Medicine*[tiab] OR Mobile Health*[tiab] OR mHealth*[tiab] OR Telehealth*[tiab] OR eHealth*[tiab] OR Health app[tiab] OR Health apps[tiab] OR Health applicat*[tiab] OR Mobile health*[tiab] OR Digital app[tiab] OR Digital apps[tiab] OR Digital apllicat*[tiab] OR “Fitness Trackers"[Mesh] OR Wearable*[tiab] OR Fitness Track*[tiab] OR Activity Track*[tiab] OR "Telerehabilitation"[Mesh] OR Telerehabil*[tiab] OR Tele-rehabil*[tiab] OR Tele rehabil*[tiab] OR Remote Rehabil*[tiab] OR Virtual Rehabil*[tiab] OR Digital health*[tiab] OR "Self Care"[Mesh] OR Self Care*[tiab] OR Self-Care*[tiab] OR "Self-Management"[Mesh] OR Self-Manag*[tiab] OR Self Manag*[tiab])

AND

("Aftercare"[Mesh] OR Aftercar*[tiab] OR After care*[tiab] OR After-Treat*[tiab] OR After Treat*[tiab] OR Follow-Up*[tiab] OR Follow Up[tiab] OR Followup*[tiab] OR Drug Usag*[tiab] OR Medication Usag*[tiab] OR Drug Use[tiab] OR Medication Use[tiab] OR Perscript*[tiab] OR "Drug Prescriptions"[Mesh] OR Prescript*[tiab] OR Prescrib*[tiab] OR "Prescriptions"[Mesh])
